# “To Have One Extra Eye”: Exploring Professionals’ Experiences with Digital Supervision in a Nursing Home for Older People

**DOI:** 10.1177/23779608261436779

**Published:** 2026-03-24

**Authors:** Helén Rönning, Sara Svanholm, Ann Ekdahl, David Johansson, Malin Holmström Rising

**Affiliations:** 1 Department of Health Sciences, 6311 Mid Sweden University, Sundsvall, Sweden; 2 Care of welfare, 191823 Nordanstig Municipality, Bergsjö, Sweden

**Keywords:** Digital technologies, gerontology, long-term care, older adult, patient safety

## Abstract

**Introduction:**

The global increase in the older population and challenges in recruiting healthcare professionals to nursing homes necessitate innovative approaches to ensuring safe healthcare delivery.

**Objective:**

To explore and describe healthcare professionalś experiences with a digital supervision system in a nursing home setting.

**Design:**

Qualitative research design.

**Methods:**

Data were collected from 28 professionals in one nursing home through seven focus group interviews and three individual interviews and were analyzed using reflexive thematic analysis.

**Results:**

The findings revealed one overarching theme: having an extra eye, which influenced work processes and patient safety. Two subthemes emerged: (1) experiences of a changed environment and stress, and (2) experiences of managing technical challenges. The digital supervision system altered professionals’ workflows, impacting both professionals and resident environments while also introducing technical difficulties. Overall, having “one extra eye” through the phone in the pocket assisted professionals in monitoring their work, and observing changes among residents contributing to an improved professional and resident's environment and preventing falls.

**Conclusions:**

This study enhances the understanding of the opportunities and challenges associated with digital supervision in a nursing home. The healthcare team continued to conduct risk assessments as before, and nurses, physiotherapists, and occupational therapists prescribed physical supervision as usual. The digital supervision system allowed professionals more time to engage in other activities with residents during the day. Being able to notice changes in residents, along with personalized alarm settings for digital supervision, may help prevent falls while still protecting residents’ independence, physical abilities, and privacy in their apartments.

## Introduction

The European Union (EU) faces a significant demographic shift, with a growing population of older people, particularly those aged 75–84. This demographic change is accompanied by an increased demand for healthcare services due to chronic diseases and severe functional disabilities, which often necessitate long-term care (LTC) (Grubanov-Boskovic et al., 2021). According to the World Health Organization (WHO), LTC encompasses activities that “ensure that people with or at risk of a significant ongoing loss of intrinsic capacity can maintain a level of functional ability consistent with their basic rights, fundamental freedoms and human dignity” (WHO, 2024, p. 8). LTC facilities, commonly referred to as nursing homes in research, provide continuous or intermittent 24-h care for older people with varying health conditions and functional abilities (WHO, 2024).

## Review of Literature

### Demand for Higher Levels of Care and a Sustainable Healthcare Workforce

As the aging population increases, the demand for higher levels of care and a sustainable healthcare workforce rises (Brady & Kuiper, 2023). However, the current workforce is insufficient to meet the EU's growing needs. When looking at the

old age dependency ratio (ODR), that gives an estimate of the proportion of the elderly population (aged 65+) in the working age population (aged 15–64). In 2019, there were 31 elderly people for every 100 people in the working age group. In 2060, this ratio would increase to 54 elderly people for every 100 persons of working age. (Grubanov-Boskovic et al., 2021, p. 31)

One of the primary reasons for the decreasing healthcare workforce is retirement, alongside an increasing number of professionals choosing to leave the sector for other fields. Factors contributing to this trend include low salaries, limited opportunities for professional development, heavy workloads, poor working conditions, and low job satisfaction. Additionally, declining birth rates affect the future availability of healthcare workers. In response to this shortage, foreign-born workers play a crucial role in maintaining the workforce. In 2018, five EU member states (Sweden, Germany, Italy, France, and Spain) reported that nearly two-thirds of healthcare and LTC workers were foreign-born (Grubanov-Boskovic et al., 2021).

With a growing number of older people requiring LTC, a shortage of healthcare professionals, and an increasing proportion of workers who do not speak Swedish as their native language, it is essential to develop new work methods. These methods must be easy to use, reduce stress and workload, enhance working conditions, and ensure patient safety (European Commission: Directorate-General for Health and Food Safety. 2021). A systematic literature review by Simmons et al. (2016) identified four key safety indicators in nursing homes: falls, pressure injuries, medication errors, and infections. However, these indicators may alone be insufficient to assess the quality of care and residents’ overall quality of life. An integrated literature review by Min et al. (2024) found that both individual and institutional factors influence the safety and well-being of older people in nursing homes. One critical institutional factor is staffing levels and the professional environment. Their study also found that the implementation of monitoring systems can alleviate staff burden while enhancing resident safety. The increasing demand for healthcare services for older people, coupled with workforce shortages and rising healthcare and social care costs, has become a major concern for policymakers and stakeholders. Welfare technologies have been proposed as a potential solution to these challenges (Baudin et al., 2020; Borg et al., 2023).

### Welfare Technologies

In Sweden, the definition of welfare technologies by the Swedish National Board of Health and Welfare in Socialstyrelsen’s term bank (2015) is “digital technology that aims at maintaining and increasing safety, activity, participation, or independence for a person who has or is at an increased risk of impairment.” This definition has been widely used in previous research (Borg et al., 2023). Previous research on healthcare professionals’ perceptions of welfare technology indicates a generally positive outlook, as it is considered an enabler that simplifies daily tasks for both care recipients and caregivers (Frennert & Baudin, 2021). However, professionals may encounter challenges when using more advanced welfare technologies due to factors such as age, language barriers, or negative attitudes toward technology (Thunberg et al., 2023). The implementation of welfare technologies is shaped by multiple perspectives, including those of older people, informal and formal caregivers, organizations, infrastructure, and technological factors. It is further influenced by facilitators and barriers such as capacity, attitudes and values, health, expectations, participation, identity, and lifestyle (Zander et al., 2021).

### Digital Supervision

One example of welfare technology is digital supervision (Swedish National Board of Health and Welfare, 2015), also referred to as digital surveillance (Frennert & Baudin, 2021), sensor technology, or passive alert systems (Nordic Welfare Centre, 2019). In this study, we use the term “digital supervision.” The choice of this term reflects the fact that professionals are not actively sitting and monitoring older people in real time; instead, the system issues alerts based on individualized settings tailored to each older person's needs, and professionals can perform a check-in when necessary using the mobile device they carry in their pocket. In Sweden, digital supervision has been identified as a promising welfare technology for care for older people, particularly in reducing nightly checkups that disturb and awaken older people (Frennert & Baudin, 2021). Evidence suggests that various strategies can help alleviate the burden on healthcare professionals (Abbe & O'Keeffe, 2021). Emilsson et al. (2023) reported that care workers using cameras especially nocturnal ones, in nursing homes emphasized the importance of support, education, and information about these technologies. They also noted improvements in the nighttime environment and the need to balance privacy with security. However, research on nocturnal digital supervision systems has shown mixed results, with some studies suggesting that they are not necessarily superior to standard care (Richardson et al., 2021). Ethical concerns, particularly regarding the autonomy, privacy, and fundamental rights of older people, remain a critical issue (Sundgren et al., 2019). Additionally, Borg et al. (2023) highlighted significant research gaps, particularly regarding professionals’ perspectives on the digital supervision system.

The increasing demand for healthcare services for older people, coupled with a shortage of healthcare professionals, presents significant challenges for LTC and care systems. Systems with digital supervision offer a promising solution for enhancing workforce sustainability and improving the efficiency and effectiveness of care for older people. However, evidence regarding the impact of digital supervision systems on routine care, how the interdisciplinary healthcare team works with the digital supervision system around the clock, and the resulting outcomes remains inconclusive. This study aims to explore and describe healthcare professionals’ experiences with digital supervision systems in nursing homes for older people. By providing insights into the practical benefits and challenges of implementing such technologies, the study contributes to workforce policies and the development of improved care strategies.

## Methods

### Design and Theoretical Framework

A qualitative inductive design with reflexive thematic analysis (Braun & Clarke, 2019) was employed to investigate professionals’ experiences with a digital supervision system. Reflexive TA was chosen as it allows for an in-depth exploration of the variations in experiences, emphasizing the dynamic and interpretative nature of meaning-making through social processes and construction (Braun & Clarke, 2019). This approach facilitated the identification of patterns and their interrelationships, providing insights into how professionals engage with and reflect on the use of digital supervision in their work.

### Setting

Nursing homes in Sweden are staffed by healthcare professionals, including nursing professionals, physiotherapists, and occupational therapists, as well as care workers, whose level of training determines their ability to meet residents’ personal needs. This study was conducted in an LTC facility for older people (nursing home) using a digital supervision system. The nursing home was in a small municipality (population: <10,000) in east-central Sweden and provided continuous 24-h care. It was operated by the municipality and consisted of 32 apartments divided into two departments. The implementation of the digital supervision system occurred in two phases. Department one had implemented a digital supervision system for approximately 2 years before data collection, whereas department two had introduced the system about 3 months prior to the study. Initially, all care workers received training provided by the company. Thereafter, 50% of the care workers underwent further training by the company to become superusers before the initiation of digital supervision.

All 32 apartments were occupied by residents with varying healthcare needs, including those related to illness, physical limitations, or cognitive impairments, such as dementia. Upon the introduction of the digital supervision system, all residents were asked to provide consent. For older people with cognitive impairments who were unable to give informed consent, licensed healthcare professionals conducted risk assessments to minimize the risk of perceived restrictive measures. These assessments followed standardized procedures established by a medically responsible nurse.

The digital supervision system registered sensor-based alarms designed to detect movement within the residents’ apartments. Care workers trained as superusers were responsible for activating and adjusting the sensor alarm settings. When the system was activated, it generated a basic layout of the common room, including the placement of the bed, living room furniture, and kitchenette. Personalized alarm settings were configured based on individual residents’ needs, determining which movements would trigger alarms. These included getting out of bed, leaving the room, moving within the room, entering the room, prolonged inactivity in bed, sound alarms, and detecting objects falling to the floor (fall alarm). Superusers monitored log controls to assess response times and ensure timely intervention. A high-priority alarm was triggered when the sensor detected an object falling on the floor, signaling a potential fall. Alerts and digital supervision were managed via an application on the professionals’ work phones. The system allowed for two levels of supervision: anonymous color mode and anonymous extended supervision (grayscale mode). In color mode, sensors registered heat signatures within the room, displaying them in color without revealing personal details. In grayscale mode, the entire room was displayed in shades of gray, offering extended supervision while maintaining anonymity.

During the day, nursing home apartments were grouped into smaller clusters. At the start of each shift, the care workers logged into their phones and selected the apartment groups for which they were responsible. Critical alarms were sent to all care workers, regardless of their assigned group, while standard alarms were directed only to personnel responsible for that specific group. At night, all 32 apartments were combined into a single group to ensure comprehensive supervision.

### Data Collection

Purposive sampling was used to select participants based on their specific experiences and knowledge relevant to the study's purpose. Professionals were eligible for interview if they were aged 18 years or older, had worked in the team at the nursing home for at least 3 months. Data was collected from February to November 2024 through seven focus group interviews and three individual interviews due to challenges in forming homogeneous groups (Nyumba et al., 2018). Individual interviews were chosen to be conducted with managers. Licensed healthcare professionals were placed in their own groups, and care workers in separate groups. The focus groups followed a mini focus group format, consisting of two to five participants per session (Nyumba et al., 2018). The nursing home manager arranged time for group interviews with the care workers. Depending on whether the care workers chose to participate or were required to work on short notice due to the realities of conducting research in a real-world setting, the number of participants per group varied. All focus group interviews were conducted in a private room within the nursing home, while individual interviews were held in digital meeting rooms.

A total of 10 interviews (seven group interviews and three individual interviews) were conducted, referred to as nr 1, nr 2, etc. The first author conducted the interviews, with the second author assisting in group interviews. The second author monitored the study, taking notes, and added follow-up questions. There were no prior relationships between the researchers and the participants. The group and individual interviews followed a semi-structured interview guide, but the conversation's flow, meaning, and interaction between the participant and researcher took precedence, in line with reflexive thematic analysis (Braun & Clarke, 2021). The interview guide included the following questions: (1) Describe an ordinary day at the nursing home working with the digital supervision system. (2) How is the system for digital supervision used in daily work with residents, and when? (3) Tell us about your experiences regarding the strengths and limitations of working with the digital supervision system. The follow-up questions were as follows: (4) Can you tell me more? (5) Can you give an example? The interviews lasted between 43 and 70 min (median: 59.5 min) and were audio-recorded. Additionally, an individual interview was conducted with a manager to gain further insights into the organizational experience of using the digital supervision system.

### Data Analysis

The group and individual interviews were transcribed into text and analyzed together as a single unit using reflexive thematic analysis (Braun & Clarke, 2006; Braun & Clarke, 2019). The process began with familiarization with the data by reading and re-reading the transcripts to develop a preliminary understanding of underlying meanings and patterns related to the aim of the study (Step 1). In Step 2, coding the data, codes were developed throughout the process, ranging from single words to short phrases (two to five words) to describe the sentences and patterns in the data. As coding progressed, a deeper understanding of the text emerged, resulting in a variety of codes. In Step 3, the codes were reviewed, and initial themes were interpreted. The first set of themes was more descriptive, closely reflecting the textual data, but these later evolved into the final themes. In Step 4, themes and codes were reflexively reviewed and refined, moving back and forth to identify patterns and variations in experiences. This process helped interpret the overarching theme of the digital supervision system, conceptualized as having one extra eye through the phone in the pocket, which influenced work processes and patient safety. In Step 5, the final themes were defined, named, and refined. This iterative process resulted in one overarching theme and two additional themes that addressed the study's aims (Braun & Clarke, 2021). In Step 6, the findings were presented.

### Ethical Considerations

This study was designed and conducted in accordance with international ethical standards (World Medical Association, 2024) and was approved on 2023.09.19 by the Swedish Ethical Review Authority in Lund, Dnr 2023-04832-01. The board determined that the study was exempt from Sweden's ethical legislation, as it did not involve highly sensitive individual data. All participants were previously informed about the confidential handling of their data and their right to withdraw from the study at any time without providing a reason. Informed consent was obtained. Data were stored separately from the participants’ names. Audio and transcribed text files were securely stored on password-protected encrypted university servers. The first author had access to the audio files, while all authors had access to the transcribed text files. The confidentiality of all participants was protected throughout the process and in the reporting of the findings.

### Rigor and Reflexivity

Several strategies were used to meet the criteria of credibility, transferability, confirmability, and reflexivity. To increase credibility, we used two methods of data collection to ensure homogeneity, which provided a good climate for the professionals to describe their experiences. The authors actively engaged in reflexivity and discussion throughout the analysis process to identify patterns and their interrelationships, describing how professionals engage with and reflect on the use of digital supervision in their work. The first author conducted the initial stages of analysis, while the other authors performed a parallel review of two interviews. The subsequent phases of analysis were completed collaboratively by all the authors until a consensus was reached. All the authors had professional and research experiences in long-term illness, nursing homes, and home care for older people. To enhance transferability and confirmability, a detailed description of the context was made and of the professionals participating in the study. Confirmability and authenticity were established by presenting the participants’ voices as citations in the results. The study adhered to the Consolidated Criteria for Reporting Qualitative Research (COREQ) checklist to ensure transparency and rigor in reporting the findings (Tong et al., 2007).

## Results

### Interview Characteristics

A total of 10 interviews (seven groups and three individual interviews) were conducted with 28 participants, see Table 1. The participants were 21 care workers, two registered nurses, one physiotherapist, one occupational therapist, and three managers closely involved in organizational operations challenges. Participants ranged in age from 22 to 63 years (median: 47 years), with 25 women and three men included. Their professional experience ranged from 1 to 30 years (median: 8.5 years), while their experience working at the specific nursing home ranged from 4 months to 30 years (median: 2.75 years). All participants had experiences of digital supervision in the nursing home between 3 months and 2 years. All care workers have been trained by the company. Additionally, four of the care workers had experience with night shifts, and five were designated as superusers of the digital supervision system. Hereafter, all participants are referred to as professionals.

**Table 1.** Participants.

Interviews	Participants	Gender	Age Range	Profession	Years Range Experiences in the Specific Nursing Home	Minutes
Nr 1 Focusgroup	2	Two women	20–25	Care workers	1–3 years	70
Nr 2 Focusgroup	3	One woman one man	35–55	Care workers	1–9 years	63
Nr 3 Focusgroup	4	Four women	35–65	Care workers	1–15 years	61
Nr 4 Focusgroup	5	Four women One man	20–65	Care workers	0.4–4 years	43
Nr 5 Focusgroup	2	Two women	40–60	Care workers	0.5–30 years	72
Nr 6 Focusgroup	5	Five women	25–55	Care workers	0.4–11 years	69
Nr 7 Focusgroup	4	Five women	30–65	2 registered nurses 1 physiotherapist 1 occupational therapist	0.5–7 years	51
Nr 8–10 Individual Interviews	One per interview	Two women One man	40–65	Managers	3–5 years	44–58

### Research Question Responses

**Figure 1.**
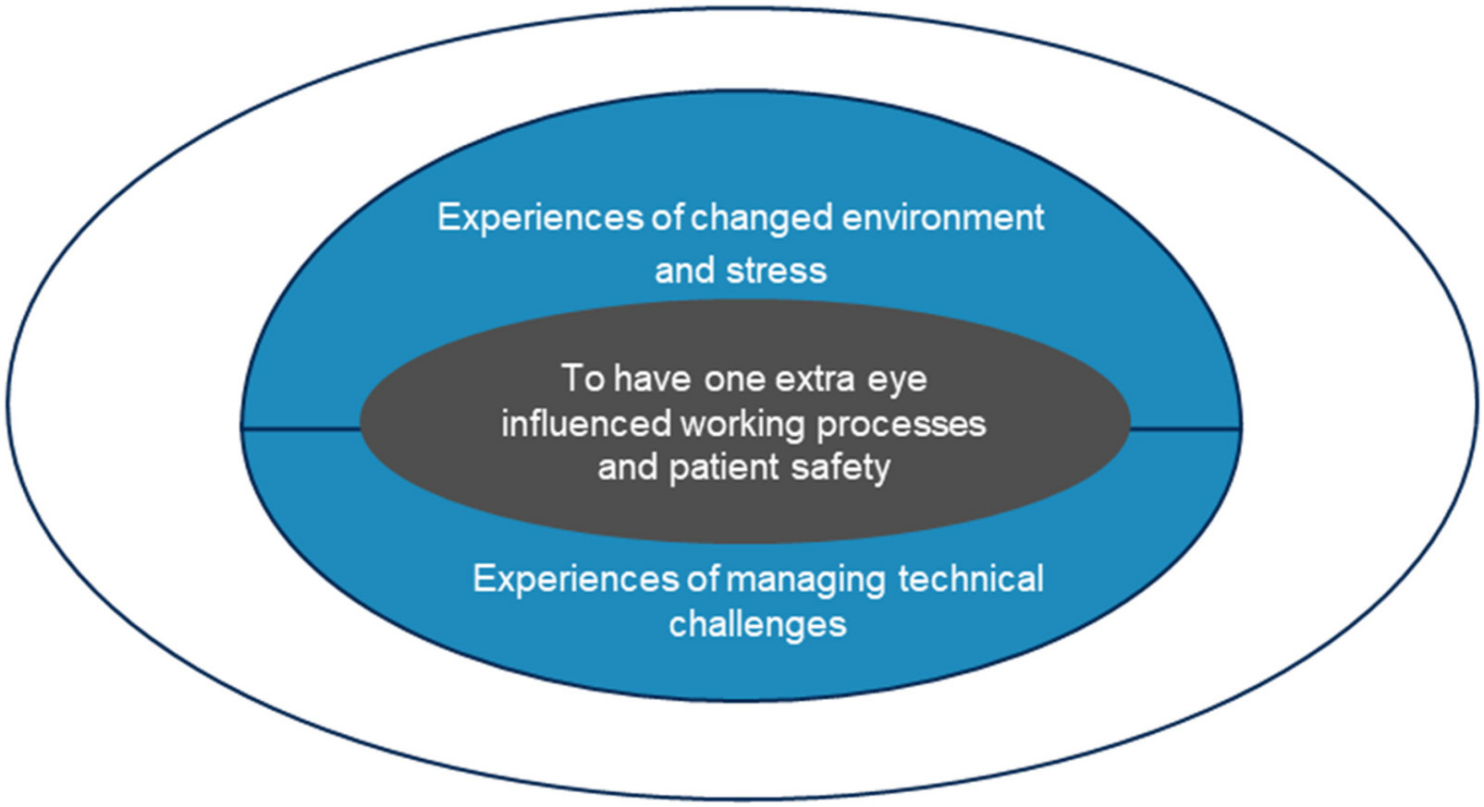
Thematic map of the finding.

The analysis revealed an overall theme: To have one extra eye influenced working processes and patient safety, with two subthemes: (1) experiences of changed environment and stress, and (2) experiences of managing technical challenges (see Figure 1).

### Theme: To Have One Extra Eye Influenced Working Processes and Patient Safety

The overall theme highlighted the concept of the digital supervision system as an extra eye through the phone in the pocket, with professionals describing how it functioned as a tool that altered their approach to working around the clock and assisted professionals in monitoring their work, “…digital supervision is a support … our extra eye … we can monitor through the system and be quicker to an acute situation such as a fall” (Nr 2). This change positively impacted on the environment of both the residents and professionals, as well as patient safety.

However, challenges were also identified, which negatively affected working processes and safety. These challenges included technical issues related to the individual settings for sensor alarms, which emerged due to evolving needs among the residents, new residents moving in, and changing professional requirements. These issues required professionals to remain vigilant, adapting to new conditions in order to maintain safety and ensure optimal care.

### Subtheme 1: Experiences of Changed Environment and Stress

This subtheme describes how professionals experienced changes in their work environment and stress levels because they had personalized alarm settings, which provided them with real-time information to prioritize their tasks.

Professionals highlighted that digital supervision, acted as an “extra eye,” allowed them to receive information that enabled them to prioritize spending time with residents instead of rushing when an alarm went off. This reduced stress and created a calmer environment. While sitting calmly in one resident's apartment or dining area, they could be assured that if anything happened in other apartments, the alarm would notify them. This allowed them to perform additional digital supervision if needed without feeling rushed.

They also described how their work environment became calmer, as the digital supervision system allowed them to focus more on personalized activities with the residents. They could attend to one resident while using digital supervision to keep an eye on others, thus feeling more present and less rushed:

…you can do something personalized with the resident and sit down in the apartment, or if someone is worried, go for a walk … yes, there is more time to spend with the resident you are with while also keeping an eye on someone who is in the bathroom by digital supervision … it allowing you to stay where you are. (Nr 6)

While the digital system offered the advantage of monitoring remotely, professionals still emphasized the importance of maintaining quality human interactions with residents. They recognized that digital supervision could not replace personal care. Depending on the alarm type and their knowledge of the resident's individual needs, they prioritized physical supervision when necessary. Another example included alarms when residents went to the bathroom. Because of their understanding of each resident's needs, professionals would often go into the room to ask if assistance was required:

When they get up from the bed, the phone rings (alarm from the system for digital supervision), and we check and wait to see if there is any alarm or anything else … otherwise, we wait … we see through digital supervision that she comes back into the room … then we leave (close digital supervision) … otherwise, if it takes three or four minutes, we go there. (Nr 1)

The introduction of the digital supervision system also improved the response time to falls. Instead of rushing to the resident's location physically, the team could now communicate and call for help through the system. Previously, it was difficult to know where other professionals were located or who had responded to an alarm, but the digital supervision system allowed for clearer coordination.

For residents who were unable to move independently or in the final stages of palliative care, professionals made decisions to deactivate parts of the digital supervision system, maintaining sensor alarms only for necessary situations. This was in line with the existing care orders for physical supervision before the digital supervision introduction.

Overall, alarms helped professionals prioritize tasks through digital monitoring, enabling residents to engage in activities in their apartments without being disturbed, which created a calmer atmosphere, “…it also becomes calmer during the day with this digital supervision … it's the same there … you don't have to attend to everything” (Nr 9). Previously, with floor sensor alarms, professionals had to repeatedly enter apartments, causing unnecessary disruptions that sometimes led to falls:

…but to know … if the person was up and walking, a floor alarm was set. This meant that professionals had to check on the person quite regularly because the alarm went off constantly … this was irritating for the person, as the professionals might have entered the room ten times in the past hour due to the floor alarm being triggered. (Nr 8)

Now, personalized alarm settings allow the residents to maintain greater independence and autonomy. In these cases, professionals found that some residents were more physically capable than originally assumed.

Professionals also noted that residents seemed to sleep better at night after the system was introduced. Rather than disturbing them to check for minor issues, such as a leg hanging off the bed, professionals were able to monitor residents more discreetly and only intervene when necessary. For residents who were easily awakened or frequently visited the bathroom, the system's settings were adjusted to minimize disruptions, ensuring they weren’t disturbed unnecessarily but also preventing falls. During the night, the team worked together more effectively, using digital supervision when required, and collaborated via the system to provide assistance to each other when needed.

Additionally, professionals reflected on how the system helped preserve residents’ anonymity. While using the system, they could not view body details but could observe movement patterns. This helped identify irregular behaviors, such as other residents visiting different departments, without breaching privacy.

### Subtheme 2: Experiences of Managing Technical Challenges

This theme focuses on how professional’s managed technical challenges associated with implementing the new digital supervision system.

At the outset of the system's installation, professionals encountered several challenges. Initially, more alarms were set up due to the learning process and the development of personalized settings, which led to a higher incidence of false alarms. Over time, however, they learned to fine-tune the system, which reduced the frequency of false alerts:

So we went from motion alarms … that is, floor alarms, to this … so, there was some uncertainty about whether this would really work … you know, a bit like that so, we took many precautions with it … yes, and set many alarms at the beginning, but … yes, now it's better. (Nr 3)

A common issue was sensor response time, where alarms were triggered either too early or too late:

…but then the alarm kept going off constantly whenever she got out of bed … it was enough for her to just move a little … sometimes just moving her arms or sitting up because her back itched and then lying down again, would trigger the alarm … and it happened frequently … it made you confused when you checked … you'd think … no, she's lying down … what's going on (Nr 5)?

Professionals reported that through trial and error, they became more adept at adjusting the alarm settings to meet each resident's personalized needs, leading to fewer false alarms.

Technical challenges also arose when the system was first implemented in the nursing home, such as when a new resident moved in or when the needs of existing residents changed. In the early stages, additional alarm settings were used until professionals became familiar with the residents and their personalized needs. Another challenge occurred when a resident had a cat. Professionals wanted to ensure that a cat jumping off the bed wouldn’t be misinterpreted as a fall or, conversely, that a fall wouldn’t be missed if the system mistook the human fall for a cat's movement.

Additionally, there were instances when the sensor couldn't detect a fall due to the angle in a room. In such cases, professionals adjusted the sensor to alert them after a period of inactivity in part of the room, compensating for the system's limitations:

…you can't see the blind spot … we had a resident who entered the blind spot and started fiddling with their refrigerator … we didn't see him … then suddenly, we wondered, where did he go … (doing physical supervision). (Nr 2)

Some residents’ changing mobility, such as transitioning between using a walker and a wheelchair, also created challenges. The professionals had to adjust the alarm settings to accommodate these shifts. Another technical challenge involved ensuring that alarm settings were communicated clearly among team members. At times, it was unclear how team should proceed with the settings or who was responsible for making the adjustments, particularly as the digital supervision system was integrated into their workflow. The team had to remain attentive and observant of changes to ensure resident safety. A specific example of this challenge arose when a resident's mobility changed, and they began using both a walker and a wheelchair within their apartment. The team had to adjust the alarm settings accordingly. A further challenge was ensuring the team could access and review alarm settings, which were recorded in a journal for care workers. To address this, written alarm settings were maintained in the paper journal, allowing authorized team members to access the necessary information. The professionals also described managing technical challenges by applying alarm settings to common situations, such as for residents without cognitive impairment, or those who could not express themselves verbally, or residents who had never experienced a fall but were at risk.

Descriptions also emerged about how log control was used in various ways to manage technical challenges. Professionals logged into the system when they checked in for work, and their activity within the alarm system was recorded, which was perceived as beneficial:

…but the alarms are also very reassuring … if something has happened, for example, if something has happened to the resident or if the resident has fallen and no one has noticed … and the alarm has gone off … you can see when the alarm went off … you can observe who received the alarm, who turned it off … then we can go directly to the source and ask, okay, what happened here? (Nr 6)

No images from digital supervision were saved, except in cases where alarms were triggered by falls. In those instances, professionals could review the situation during the alarm. They had to request access from the system owner within 24 hr; otherwise, the data would be deleted. Another technical challenge was that professionals had to manually check in and out of the apartment each time they entered or exited. If the professionals did not check in, the sensor alarm was triggered by the professionals’ movements, based on the residents’ alarm settings. However, professionals could not be registered unless they perform the check-in. If they missed checking out, the sensor alarm in the apartment would be deactivated until further observation:

…and you have to remember to leave, otherwise there won't be an alarm if you forget … and it's very important to remember … it's easy to go and do something else … throw away some trash or something, and have the phone in your pocket while it's on … so, maybe it's a bit negative, it should pop out after a while. (Nr 4)

They could check the inside and outside of the apartment directly through the app on their phone or at the sensor when entering or exiting the room. To check out, they needed to “pause” at the door; if they did not pause long enough, the checkout would not go through. They also described how they established reminders to ensure that professionals had checked in and out. Other technical challenges that were not fully addressed involved situations where professionals had impaired vision or difficulty hearing or seeing the alarm on their phone.

Additionally, professionals noted that the digital supervision system became less visible to residents over time. The device, which was mounted on the wall of the resident's apartment, might have gone unnoticed by the residents as time passed. Although the professionals had informed the residents about the system, they acknowledged that residents were likely to forget about it, “…it's not just once, but it can be every time you come in as it can change for someone with cognitive impairment” (Nr 10).

## Discussion

The results of the present study explored and described the healthcare teams’ experiences with a digital supervision system within a nursing home for older people. To the researcherś knowledge, this study may be among the first to examine healthcare teamwork processes when using digital supervision around the clock and to investigate how professionals experienced its impact on both their work and the older person.

The findings show that having an “extra eye” accessible via a phone in the pocket supported professionals in monitoring their work and contributed to changes in work processes and patient safety. Technical challenges emerged as part of the learning process and needed to continuously be adapted to the residents’ changing needs, but these challenges were addressed accordingly. The healthcare team continued to conduct risk assessments as usual, and registered nurses, physiotherapists, and occupational therapists prescribed physical supervision in their customary manner, for example, in palliative care. The personalized alarm settings within the digital supervision system resulted in fewer false alarms. When an alarm was triggered, the system provided sufficient information for professionals to determine whether they should remain with the residents or initiate physical supervision. This was perceived as reducing stress and promoting a calmer work environment. These positive changes in the environment align with the benefits reported by Emilsson et al. (2023); Frennert & Baudin, 2021 and Frennert et al. (2024).

The results also demonstrated how professionals were able to care for residents’ independence and respect their autonomy by performing digital supervision remotely, in line with the findings of Frennert et al. (2024). One of the most surprising results was how the system for digital supervision, according to the professionals, positively influenced the working processes in everyday work near the older person, which was perceived to positively affect the resident's situation round-the-clock and to support fall prevention. These results are supported by those of Min et al., (2024), who identified that institutional factors affected safety, and that professionals’ work environment and the use of monitoring systems can reduce their burden and enhance residents’ safety.

Furthermore, the results confirmed previous studies by Emilsson et al. (2023) and Frennert et al. (2024) regarding the positive impact of digital supervision at night. It not only improved the working conditions for professionals but also enhanced the residents’ experiences, leading to calmer work situations, more opportunities for collaboration, reduced physical supervision, and better sleep for the residents (Emilsson et al., 2023; Frennert et al., 2024).

Frennert et al. (2024) found technical problems to be inconvenient and described various challenges related to handling welfare technology, such as the need for charging, battery replacement, dirty robotic tools, and managing residents who were emotionally dependent on robotic dolls. In contrast, the present study focused on digital supervision, and the results showed technical challenges and how working processes and patient safety evolved. The results also identified technical challenges, such as user needs, including professionals with impaired vision, difficulty in seeing alarms, and challenges in performing digital supervision via mobile phones. Surprisingly, the professionals did not describe experiences of stress related to these technical challenges. Frennert et al. (2024) found that digital technology could disrupt workflows, leading to delays, inefficiencies, stress, and additional invisible work. However, these results were not found in the present study. One possible explanation could be that the digital supervision system was integrated into existing workflows and replaced the previous alarm system rather than being an additional task, thereby preventing it from being perceived as extra work. Additionally, systems for digital supervision that include documentation functions exist, but this feature was not used in this study. Another reason why increased stress was not reported could be that technical challenges primarily occurred when a new resident moved in. The challenges that arose due to changes in residents’ conditions seemed to develop gradually, which may have allowed professionals to adapt over time. Therefore, these challenges may not have been perceived as stressful or significantly impacting the workload.

Other studies have described technical challenges during the implementation of a digital supervision system, including a learning period. In this study, the results also found descriptions of the learning period in routine care with the digital supervision system when new residents move into a nursing home or when residents’ needs change. The results showed how professionals emphasized the need for the team to always remain attentive in routine care to observe changes and maintain patient safety.

There were also experiences where teamwork processes and the division of responsibilities among professionals were sometimes unclear due to the digital supervision system. A positive aspect was how the organization adapted and reorganized when challenges arose, which may have mitigated potential stress, as it was not explicitly described in the findings.

Stress of conscience is defined as “a product of the frequency of the stressful situation and of the perceived degree of troubled conscience as rated by healthcare personnel themselves” (Glasberg et al., 2006, p. 636). Jokwiro et al. (2022), identified in a scoping review five themes summarizing factors associated with stress of conscience: demographic factors, burnout, moral sensitivity, workplace stress, and quality of care. Protection against stress of conscience included a supportive environment, mastery and control of work duties, reduced conflict between colleagues, occupational belonging, and reflective opportunities for healthcare workers facilitated by trained personnel. Additionally, interventions such as implementing guidelines for improving person-centered care, increasing quality time with clients, stress management courses, and co-designed interventions using participatory action research were found to help reduce stress of conscience (Jokwiro et al., 2022). In the present study, the results showed how professionals adapted their working methods, allowing them more time with residents and personalized activities. This was associated with a calmer working environment, which may have had a positive impact on their experience of stress of conscience. However, challenges arose when residents forgot about the digital supervision system in their apartments, potentially leading to some level of stress of conscience among professionals. Sundgren et al. (2019) found that safety served as a counterbalance to the basic rights of privacy and autonomy of older people. These ethical challenges were not identified in the present study results, but may exist. Sundgren et al. (2019) also found fear of losing human contact and social interactions. Ethical challenges may not have been found because the present study focused on how professionals used the digital supervision system, and most interviews were conducted in groups, which may have limited such reflections. Some results indicate that professionals can experience conflicting feelings about using digital supervision, potentially causing moral distress. Creating spaces for professionals to express their thoughts and emotions is important in nursing homes. To increase ethical awareness and reduce the eventual stress of conscience, ethical dialogue as reflective opportunities for healthcare professionals should be provided (Jakobsen et al., 2023).

### Strengths and Limitations

This study has several strengths and limitations. Since social processes and constructions influence how a phenomenon is experienced, group interviews were conducted. However, due to challenges in forming homogeneous groups (Nyumba et al., 2018), individual interviews were chosen to be conducted with managers. To also support autonomy and minimize hierarchical influence in group interviews, licensed healthcare professionals were placed in their own groups, and care workers in separate groups. A key strength of this study is that the participants were healthcare professionals from the care team surrounding the resident's day and night, who were directly or indirectly affected by and working with the digital supervision system. Another strength was the use of a reflexive thematic analysis that was chosen to address the research aim. Reflexivity in the analytical process effectively captured patterns and their interrelations, contributing to a deeper understanding of the research findings (Braun & Clarke, 2019). Because the study was conducted in a real-world setting, interview timing could not always be controlled. Data collection occurred over 9 months, which led to variations in descriptions as digital supervision evolved over time. A follow-up interview with one participant was conducted to gain deeper insights into emerging patterns and increase the credibility of the study. Before and during data collection, researchers anticipated uncovering negative experiences related to being monitored while working under a digital supervision system. However, the participants reported that although they initially had concerns about digital supervision, they did not experience these concerns after using the system. Information power and saturation were not explicitly considered. The analysis was concluded when the authors determined that the research aim had been achieved (Step 5) (Braun & Clarke, 2021). The study was conducted in a nursing home within a single municipality in Sweden, which may affect the transferability. Although nursing homes in Sweden vary in terms of size, organization, and demographics, the professions in the team are typically the same as in this study. The findings might be transferable to LTC in similar contexts (Braun & Clarke, 2006).

### Implications for Practice

This study contributes to the understanding of how digital supervision systems can enhance work processes in the healthcare team, improve patient safety, and create a better environment for both professionals and residents. A key finding was that digital supervision systems may allow professionals more time to engage in other activities with residents during the day. Personal presence was not replaced by digital supervision in palliative care, and registered nurses, physiotherapists, and occupational therapists carried out risk assessments to prevent restrictive measures in the usual way, even when digital supervision was introduced. Additionally, personalized alarm settings for digital and remote digital supervision may help prevent falls while preserving residents’ autonomy and physical capabilities by ensuring privacy in their apartments. To support professions in addressing ethical aspects related to digital supervision, structured ethical dialogue should be incorporated into routine practice.

## Conclusion

Digital supervision systems appear to positively impact residents’ situations, professionals’ work environments, and patient safety. These benefits were observed around the clock. Further research is needed to evaluate resident perspectives, ethical considerations, and the broader impact of digital supervision on the working environment, patient safety, fall prevention, and health economics.

## Supplemental Material

sj-pdf-1-son-10.1177_23779608261436779 - Supplemental material for “To Have One Extra Eye”: Exploring Professionals’ Experiences with Digital Supervision in a Nursing Home for Older People Supplemental material, sj-pdf-1-son-10.1177_23779608261436779 for “To Have One Extra Eye”: Exploring Professionals’ Experiences with Digital Supervision in a Nursing Home for Older People by Helén Rönning, Sara Svanholm, Ann Ekdahl, David Johansson and Malin Holmström Rising in SAGE Open Nursing

sj-docx-2-son-10.1177_23779608261436779 - Supplemental material for “To Have One Extra Eye”: Exploring Professionals’ Experiences with Digital Supervision in a Nursing Home for Older People Supplemental material, sj-docx-2-son-10.1177_23779608261436779 for “To Have One Extra Eye”: Exploring Professionals’ Experiences with Digital Supervision in a Nursing Home for Older People by Helén Rönning, Sara Svanholm, Ann Ekdahl, David Johansson and Malin Holmström Rising in SAGE Open Nursing
