# Supplementary material for: “To Have One Extra Eye”: Exploring Professionals’ Experiences with Digital Supervision in a Nursing Home for Older People
Source: SAGE Open Nurs. 2026 Mar 24;12:23779608261436779. doi: 10.1177/23779608261436779 (PMC13014000; doi:10.1177/23779608261436779)
Supplement: sj-docx-2-son-10.1177_23779608261436779 - Supplemental material for “To Have One Extra Eye”: Exploring Professionals’ Experiences with Digital Supervision in a Nursing Home for Older People [file sj-docx-2-son-10.1177_23779608261436779.docx]

Supplementary file

Interview guide

1) Describe and ordinary day at the nursing home working with the digital supervision system.

2) How is the system for digital supervision used in daily work with residents, and when?

3) Tell us about your experiences regarding the strengths and limitations of working with the digital supervision system.

Follow-up questions:

Can you tell me more?

Can you give an example?
